# Tobacco Use Decreases Visual Sensitivity in Schizophrenia

**DOI:** 10.3389/fpsyg.2018.00288

**Published:** 2018-03-06

**Authors:** Thiago M. P. Fernandes, Michael J. Oliveira de Andrade, Jessica B. Santana, Renata M. Toscano Barreto Lyra Nogueira, Natanael A. dos Santos

**Affiliations:** ^1^Department of Psychology, Federal University of Paraiba, João Pessoa, Brazil; ^2^Perception, Neuroscience and Behavior Laboratory, Federal University of Paraiba, João Pessoa, Brazil

**Keywords:** schizophrenia, perception, visual sensitivity, tobacco addiction, smoking, public health

## Abstract

Smoking prevalence in patients who are diagnosed with schizophrenia (SCZ) is higher than in the general population. Chronic tobacco use in SCZ patients may reduce the side effects of antipsychotic drugs, thus serving as a self-medication for such side effects. Understanding the ways in which chronic tobacco use influences visual sensitivity has clinical implications, which may serve as a tool for non-invasively diagnosing early-stage visual processing deficits. The present study evaluated the effects of chronic tobacco use on visual sensitivity in SCZ patients. Our purpose was to provide new directions for future research, mainly psychophysical and electrophysiological studies. In the present study, 40 smoker controls (SC), 20 SCZ tobacco users, and 20 SCZ tobacco nonusers were recruited from the Psychosocial Care Center. Visual sensitivity was compared between both SCZ groups and the SC group. Patients with SCZ who were chronic tobacco users presented lower visual sensitivity for chromatic (*p* < 0.001) and achromatic (*p* < 0.001) stimuli compared with the other groups. Our findings highlight the need to evaluate possible addictive behavior in patients with SCZ, which may contribute to public policies that seek to improve the quality of life of SCZ patients and their families.

## Introduction

Schizophrenia (SCZ) is a chronic, disabling, and debilitating disorder of the central nervous system that includes both psychotic and cognitive symptoms. It affects 1.0% of the population worldwide and is a public health concern (Mathers and Loncar, 2006; Jablensky, 2010). Patients with SCZ may have comorbidities, such as depression, anxiety, and substance abuse (Buckley et al., 2009; Sagud et al., 2009).

Studies that investigated substance abuse in patients with psychiatric disorders suggest that tobacco abuse may act as a form of self-medication for disease distress (Manzella et al., 2015). Chronic smoking is oftentimes comorbid with SCZ, which can affect visual sensitivity. Studies reported that patients with SCZ have a high rate of tobacco use and can be classified as heavy tobacco users (i.e., ≥25 cigarettes per day compared with <20% of the general population who smokes; Kelly and McCreadie, 2000). Patients who are diagnosed with SCZ may have a higher intensity of tobacco use and a lower rate of cessation compared with the general population (Beck et al., 2015).

Tobacco cigarettes contain numerous compounds that are harmful to health (World Health Organization [WHO], 2015). The most common form of cigarette absorption is through the main alkaloid in the cigarette, nicotine. Nicotine is a psychoactive ingredient in cigarettes that quickly reaches the brain through the airways (Balfour and Munafò, 2015). Nicotine binds to nicotinic acetylcholine receptors (nAChRs) in the central and peripheral nervous systems and upregulates the number of nAChRs (Levin, 2001; Metherate, 2004; Govind et al., 2009). The greater number of nAChRs is related to a greater need to use tobacco, thus strengthening addiction (Dani and De Biasi, 2001; D’Souza and Markou, 2011).

Schizophrenia is characterized by an imbalance of neurotransmitters, especially dopamine hyperfunction, and acetylcholine is involved in the ability of nAChRs to regulate dopaminergic activity (Levin, 2001; Blake et al., 2015). The activation of nAChRs increases dopamine release, and nicotine interacts with dopaminergic neurons that have cortical and subcortical projections (Levin, 2001; Balfour and Munafò, 2015).

Regardless of smoking status, SCZ patients present impairments in visual information processing, including motion perception, perceptual organization, spatial localization, temporal prediction, eye tracking, color perception, and visual contrast sensitivity (Yee et al., 1987; Shuwairi et al., 2002; Butler et al., 2008; Levy et al., 2010; Herzog and Brand, 2015; Martin et al., 2017). The results of studies that investigated changes in visual sensitivity in SCZ have been controversial. Generally, some authors argue that an imbalance in neurotransmission is responsible for a decrease in visual ability (Kéri et al., 2002; Chen et al., 2003; Cadenhead et al., 2013). Others argue that molecular dysfunction of visual processing pathways is both a cause and effect of visual impairment (Martínez et al., 2008; Gracitelli et al., 2013). As stated, losses in visual sensitivity in SCZ have been discussed in the literature (Silverstein and Rosen, 2015), indicating the existence of possible dysfunction of the magno- and parvo pathways. The M-pathway is highly sensitive to low spatial frequencies, whereas the P-pathway is highly sensitive to medium and high spatial frequencies. For example, Martínez et al. (2008) used functional magnetic resonance imaging and found that patients presented a decrease in M-pathway activity and a consequent decrease in the detection of low spatial frequencies (observed in the parietal and temporal lobe). When dysfunction of these pathways occurs, there may be losses in signal amplification, as reported by Kéri et al. (2000), Butler et al. (2005), Chen (2011), Schallmo et al. (2015), and others. This dual channel is further addressed in the section “Discussion.” However, other results suggest that overall dysfunction may be a more plausible approach than specific dysfunction of, for example, only the M-pathway (Herzog et al., 2013; Herzog and Brand, 2015). For a broad discussion between the magno- and parvocellular pathways, see Skottun (2015).

Likewise, heavy tobacco users without neuropsychiatric disorders, regardless of gender or age, also present spatiotemporal impairments (Kunchulia et al., 2014; Fernandes et al., 2017a). One may argue that this would occur in heavy tobacco users with SCZ. However, we need to consider that SCZ patients may use tobacco chronically for self-medication. The use of cigarettes in this population may reduce adverse effects or improve symptoms (Bakanidze et al., 2013). Investigating visual processing in patients with SCZ may serve as a noninvasive diagnostic tool to improve prognosis and quality of life (Silverstein et al., 2015).

The contrast sensitivity function (CSF) is a classic measure that was developed in the 1960s (Campbell and Maffei, 1974) to describe the mechanisms that are involved in spatial processing. The CSF is one of the main measures to verify the response of the visual system in healthy, preclinical, and clinical populations under scotopic, mesopic, and photopic luminance conditions. The CSF also allows observations of activity of the magnocellular, parvocellular, and koniocellular visual pathways (Xu et al., 2001; Ahmadi et al., 2015; Skottun, 2015). It also allows determination of the response of the visual system in processing rough attributes and fine details of objects that are modulated by low, medium, and high spatial frequency bands (DeValois and DeValois, 1990; Cornsweet, 2012).

Color vision-related losses can be either acquired or congenital (Lanthony and Dubois-Poulsen, 1973). Color vision loss can result from optical, neural, or systemic disease (Simunovic, 2016). Conditions that affect the nervous system, such as SCZ, may affect color vision (Nork, 2000; Costa et al., 2007; Fernandes et al., 2017b), ranging from ophthalmic diseases to pathology of the visual cortex. There is a large gap in the literature with regard to studies that evaluated color vision in SCZ. New tools and further studies are needed to completely assess this system. To avoid within-subject variability, the Cambridge Colour Test (CCT) allows the precise control of chromaticity parameters of stimuli, multiple randomized presentations of initial target-background chromaticity differences, and a staircase psychophysical procedure for estimating discrimination thresholds (Paramei, 2012). Thus, only one experimental session is sufficient to characterize color detection thresholds because repeated measurements are used. As one test of the CCT, the Trivector test is another way to assess congenital and acquired damage and can be performed quickly (i.e., <5 min). The advantage of a computer-controlled test is that the difference between the stimulus and background can be adjusted dynamically according to individual performance (Mollon and Regan, 2000). However, the Trivector test uses pseudoisochromatic stimuli that exhibit dynamic variations in chromaticity between the target and its background (Hasrod and Rubin, 2015; Fernandes et al., 2017c).

Considering these shortcomings, the main purpose of the present study was to investigate the effects of tobacco use on visual processing in patients who were diagnosed with SCZ. Understanding the relationship between visual processing and comorbid tobacco use and SCZ may have clinical applications. The present study evaluated the effects of tobacco use on visual sensitivity in SCZ patients. Our hypothesis was that chronic cigarette use affects the retina-geniculate-striate system through the inhalation of toxic smoke or the absorption of health-damaging particles that are found in cigarettes. We expected that tobacco users with SCZ would present greater impairments in visual sensitivity than SCZ patients who did not use tobacco.

## Materials and Methods

### Participants

Forty smoker controls (mean age = 35.7 years; *SD* = 7.87 years), 20 SCZ patients who were chronic tobacco users (mean age = 34.6 years; *SD* = 8.39 years), and 20 SCZ patients who did not use tobacco (mean age = 32.3 years; *SD* = 5.39 years) were recruited. The smoker control (SC) group comprised staff or students at the Federal University of Paraiba who were recruited through newspaper advertisements. Patients who were diagnosed with SCZ were recruited from the Psychosocial Care Center in Paraiba. Psychiatrists at the same institution used the criteria of the *Diagnostic and Statistical Manual of Mental Disorders*, 5th edition (DSM-5; American Psychiatric Association, 2013), to diagnosis SCZ. We recruited patients who used atypical antipsychotics because this class of antipsychotics causes less impairment in spatial vision than typical antipsychotics (Chen et al., 2003; Cadenhead et al., 2013). Antipsychotic dosages were transformed into chlorpromazine-equivalent dosages to avoid differences between dosages. With regard to assessing visual sensitivity, we controlled for weight and the duration of medication use, for which no statistically significant differences were found between groups. We sought to minimize confounding factors, such as high dosages of antipsychotics, which can cause weight gain (Bak et al., 2014) and metabolic syndrome and affect eye health (Cheung and Wong, 2007; Poh et al., 2016). The SCZ patients used the following antipsychotics: quetiapine (*n* = 17), olanzapine (*n* = 14), risperidone (*n* = 7), clozapine (*n* = 1), and ziprasidone (*n* = 1). The patients were taking antipsychotic monotherapy. For patients who used benzodiazepines (12% of the sample), we stipulated that the maximum dosage should be 20 mg/day (diazepam dose equivalent). They were instructed not to use benzodiazepines for 3 days prior to the experiment to avoid possible residual effects within the drugs’ half-life. Similar results were obtained when patients treated with benzodiazepines were excluded from the analysis, and here we report the results in the whole group of patients.

All of the tobacco users met the criteria for tobacco use disorder according to the DSM-5. They currently smoked >20 cigarettes/day and had a score >7 on the Fagerström Test for Nicotine Dependence (FTND; Heatherton et al., 1991). The control group was composed of smokers who had no additional neuropsychiatric disorders according to the Structured Clinical Interview for the DSM (American Psychiatric Association, 2015).

Subjects were excluded if they met any of the following exclusion criteria: <25 years old or >45 years old, current history of neurological disorder, cardiovascular disease, history of head trauma, history of contact with such substances as solvents, current or previous drug abuse, and current use of medications that may affect visual processing and cognition. Female participants who used oral contraception were only tested outside their menstrual period to minimize possible confounds of hormonal differences. However, recent findings support the notion that the menstrual cycle does not affect contrast sensitivity (Webb, 2016).

The subjects were required to have good ocular health, with no abnormalities on fundoscopic or optical coherence tomographic examination. An ophthalmologist had examined them during the last 12 months. All of the participants were screened for color blindness using Ishihara’s (1972) tests for color deficiency and had normal or corrected-to-normal vision as determined by visual acuity of at least 20/20, measured by the Snellen eye chart.

The time since last cigarette use was assessed by a self-report to equate withdrawal across tobacco users. All of the subjects were asked to abstain from caffeine-containing products beginning at 12:00 AM the evening prior to the measurements. No significant differences in depression and anxiety symptoms were found before and after the study, measured by the Hamilton Scale for Depression and Hamilton Anxiety Rating Scale. Both groups were matched for gender, age, and level of education. The subjects participated in the study on a voluntary basis.

The present study followed the ethical principles of the Declaration of Helsinki and was approved by the Committee of Ethics in Research of the Health Sciences Center of Federal University da Paraiba (CAAE: 45774715.9.0000.5188). Written informed consent was obtained from all of the participants.

### Visual Contrast Sensitivity

#### Stimuli and Apparatus

Stimuli were presented on a 19-inch LG CRT monitor with 1024 × 786 resolution and a 100 Hz refresh rate. Stimuli were generated using a VSG 2/5 video card (Cambridge Research Systems, Rochester, Kent, United Kingdom), which was run on a Precision T3500 computer with a W3530 graphics card. The average luminance was 50 cd/m^2^. All of the procedures were performed in a room at 26°C ± 1°C. The walls of the room were covered in gray to better control luminance during the experiments. Measurements were performed with binocular vision at a distance of 150 cm from the computer monitor. The luminance of the monitor and chromatic calibrations were performed using a ColorCAL MKII photometer (Cambridge Research Systems, Rochester, Kent, United Kingdom).

##### Contrast sensitivity function

The contrast sensitivity measurements were taken using Metropsis software (Cambridge Research Systems, Rochester, Kent, United Kingdom). This software provides a clinical evaluation of the CSF. The Metropsis vision-testing suite provides precise, repeatable, psychophysical threshold measurements for general research applications. Stimuli for the CSF were linear and vertically oriented and had sine wave gratings with spatial frequencies of 0.2, 0.6, 1.0, 2.0, 5.0, 10.0, and 20.0 cycles per degree (cpd). The stimuli consisted of equiluminant gratings with dimensions of 5 degrees of visual angle, which were presented on the monitor at 2.5° spatial offset from the central cross-shaped fixation point (for stimulus details, see dos Santos et al., 2013; Fernandes et al., 2017a).

##### Color vision

The CCT provides a clinical assessment of color vision deficiencies as a rapid means of screening the presence of congenital or acquired deficits (Mollon and Regan, 2000). The CCT uses pseudoisochromatic stimuli (Landolt C), defined by test colors that are to be discriminated, on an achromatic background. The figure and background are composed of grouped circles that randomly vary in diameter and have no spatial structure (variation of 5.7° arcmin of external diameter and 2.8° arcmin of internal diameter). The luminance variation in each response avoids possible learning effects or guessing to respond correctly. The Trivector testing protocol estimates sensitivity to short, medium, and long wavelengths through protanopic, deuteranopic, and tritanopic confusion axes, respectively (Regan et al., 1994; Mollon and Regan, 2000). The Trivector protocol uses vectors as a central measurement. The advantage of this brief test is that it can be performed in approximately 5 min and provides reliable results. The three confusion axes converge at a co-punctual point. The following u’v’ coordinates (Mollon and Regan, 2000) were used: protan (0.6579, 0.5013), deutan (-1.2174, 0.7826), and tritan (0.2573, 0.0000; for more details, see Mollon and Regan, 2000).

In general, we used a default setting in which the Landolt C had an opening at 1° of visual angle, minimum luminance of 8 cd/m^2^, maximum luminance of 18 cd/m^2^, 6 s of response time life for each trial, and distance of 269 cm between the participant and the computer monitor.

### Cognitive Measures

#### Stroop Color-Word Interference

The Stroop Color-Word Interference test (Proctor, 1978) was used to measure executive function, such as attention, cognitive flexibility, inhibition, and information processing speed. A series of color words was presented to the participants, and their task was to name the color of each word that was presented. We used four colors (red, blue, yellow, and green) in several combinations that were randomly displayed on a computer screen. The measure was the number of elements that were properly named. Fewer errors with incongruent stimuli indicated better performance.

#### Flanker Task

The Flanker Task (Eriksen and Eriksen, 1974) was used to evaluate attentional control and inhibition. In the task, stimuli (letters, such as ZXYQ) were centrally presented and flanked by peripheral stimuli. We used reaction time as a measure of cognitive ability. A quicker reaction time indicated better performance.

### Procedure

The procedures were performed in two stages. In the first stage, the participants were referred to our laboratory where we conducted the cognitive tasks. A specialist performed the neuropsychological tests. This procedure was performed in a quiet and comfortable room that was dedicated to the experiment. The approximate time of the experiment was 1 h 30 min for each participant. In a second meeting, the participants underwent the visual measurements. Each session of the second stage lasted 30–45 min. For all of these procedures, the participants were encouraged to take breaks between each block of measurements to avoid fatigue.

#### Contrast Sensitivity Function

The two-alternative forced-choice (2-AFC) method was used, and the subjects’ task was to identify, using a remote control response box, whether the stimulus was presented on the left or right side of the monitor. The participants were also instructed to respond whether they could not identify the stimuli because Metropsis software uses a psychometric function that describes thresholds without interference from guessing. The subjects were instructed to maintain fixation on a small black fixation cross in the center of the display monitor. The order of the spatial frequencies that were tested was randomized within a session.

A three-down one-up logarithmic staircase with dynamic steps was used, tracking performance at 79% accuracy (Levitt, 1971). Initially, the contrast values appeared at a suprathreshold level (for which the participants were expected to emit a series of correct answers) to reach the staircase criteria of three consecutive correct responses and an error. After three consecutive correct responses, contrast decreased by 0.7 dB. After every incorrect response, contrast increased by 1.0 dB. Each stimulus had an exposure time of 600 ms with a 300 ms intertrial interval. This procedure was conducted throughout the experiment.

To obtain the reversals for each grating, an average of 150–250 trials (depending on the participant) was presented. The session ended after 12 reversals that were recorded for each grating that was tested. Contrast levels for all reversals, except for the first two (motivation effect) and last two (fatigue effect), were sufficient to produce a contrast detection threshold. Higher contrast sensitivity values indicated that the participant presented higher sensitivity to the spatial frequency that was evaluated in the test (for procedural details, see dos Santos et al., 2013; Fernandes et al., 2017a). The participants were encouraged to take breaks at their discretion to avoid fatigue.

#### Color Vision

The four-alternative forced-choice (4-AFC; Jäkel and Wichmann, 2006; Paramei, 2012) method was used. The subjects’ task was to identify, using a remote control response box, the position of the opening/gap in the Landolt C stimulus. The participants were instructed to answer even if they could not identify the stimulus gap (Mollon and Regan, 2000). After each correct answer, the chromaticity of the target proceeded closer to the chromaticity of the background. Each wrong answer or omission was followed by the presentation of the target at a greater chromatic distance from the background. The step on the staircase was doubled or divided by two after each incorrect or correct answer, respectively. This procedure was conducted throughout the experiment. The experiment ended after 11 reversals for each axis, and the threshold was estimated from the six final reversals (Mollon and Regan, 2000; Paramei and Oakley, 2014).

### Clinical Measures

To assess subjective craving for cigarettes, the participants provided self-reports and completed psychometric measures, including the Program to Aid Smokers (PAS) comfort scale (Issa, 2012) and the brief version of the Questionnaire of Smoking Urges (QSU-B; Cox et al., 2001). The Young Mania Rating Scale (YMRS; Young et al., 1978) and Brief Psychiatric Rating Scale (BPRS; Leucht et al., 2005) were used to determine mood state and illness severity.

### Statistical Analysis

The statistical analysis was performed using SPSS 23.0 software. The data distributions were assessed for normality using the Shapiro–Wilk test. The data from both groups presented a non-normal distribution; thus, nonparametric statistical tests were used to analyze the data. Homogeneity and sphericity were not violated. Visual contrast sensitivity deficits among the SCZ groups were determined using tolerance limits based on the SC group’s visual sensitivity thresholds. The Kruskal–Wallis test was used, followed by the Mann–Whitney *U*-test.

Nonparametric regression analyses (bootstrapping) were performed to evaluate relationships between clinical measures, cognitive performance, and visual sensitivity between groups.

The effect size (*r*) was estimated based on z-score conversion (Field, 2013). Effect sizes >0.50 were considered medium-to-large effect sizes. Values of *p* < 0.016 were considered statistically significant.

## Results

### Sample Characteristics

The sample characteristics of the participants are summarized in **Table 1**. The groups did not differ in age ($\chi_{2}^{2}$ = 3.591, *p* = 0.166), level of education ($\chi_{2}^{2}$ = 6.723, *p* = 0.035), or the ratio of males to females ($\chi_{2}^{2}$ = 0.296, *p* = 0.862). The tobacco users had an average score of 7 on the FTND. No differences in scores on the BPRS (*U* = 368, *p* = 0.620), Hamilton Rating Scale for Depression (*U* = 267, *p* = 0.141), and Hamilton Anxiety Rating Scale (*U* = 188, *p* = 0.583) were found between the SCZ groups. No statistically significant differences were found between SCZ tobacco-users before (*M* = 26.33, *SD* = 4.0) and after (*M* = 25.11, *SD* = 0.05) experiment as measured by QSU-B scores (*p* = 0.644).

**Table 1:** Sample characteristics.

Variable	SC (*n* = 40)	SCZ tobacco non-users (*n* = 20)	SCZ tobacco users (*n* = 20)	*p*
**Gender**				
Male	29	13	15	0.046^a^
Female	11	7	5	0.671^a^
**Age**				
Age, years (*SD*)	35.4 (8.0)	35.1 (8.1)	38.4 (7.5)	0.569^b^
**Level of education, years (*SD*)**	10.6 (2.4)	9.3 (2.1)	8.5 (2.5)	0.652^b^
**Age of onset, years (*SD*)**	–	19.8 (3.1)	22 (2.0)	0.599^#,b^
**Duration of illness, years (*SD*)**	–	10.5 (2.5)	12.3 (3.1)	0.746^#,b^
**Number of hospitalizations**	–	6 (2)	5 (4)	0.891^#,b^
**Young Mania Rating Scale score**	–	6.1 (6.1)	4.6 (3.1)	0.491^#,b^
**Brief Psychiatric Rating Scale score**	–	41.8 (6.7)	44.1 (4.8)	0.405^#,b^
**Program to Aid Smokers Comfort Scale score**				
Before experiment	–	–	18.14 (4.3)	N/A
After experiment	–	–	17.11 (2.0)	N/A
**Questionnaire of Smoking Urges-B score**				
Before experiment	–	–	26.33 (4.0)	N/A
After experiment	–	–	25.11 (0.5)	N/A
**Chlorpromazine equivalent dose (mg)**	–	364.22 (190)	415.81 (114)	0.333^#,b^
**Diazepam equivalent dose (mg)**	–	3.25 (1.5)	4.05 (2.3)	0.067^#,b^

*^#^Between schizophrenia (SCZ) groups, ^*a*^χ^*2*^ test, and ^*b*^Student’s t-test.*

With regard to the cognitive assessments, the Kruskal–Wallis *H*-test revealed a statistically significant difference in Flanker task scores between groups ($\chi_{2}^{2}$ = 8.64, *p* = 0.013, *w*^2^ = 0.109), with a mean score of 27.34 in the SC group, 28.63 in the SCZ tobacco-nonuser group, and 38.75 in the SCZ tobacco-user group. Pairwise comparisons showed that SCZ tobacco users exhibited impairments in the Flanker task compared with the SC group (*p* < 0.001) and SCZ tobacco-nonuser group (*p* = 0.008). No differences in Stroop task performance were observed between groups ($\chi_{2}^{2}$ = 4.37, *p* = 0.113).

### Contrast Sensitivity Function

**FIGURE 1:**
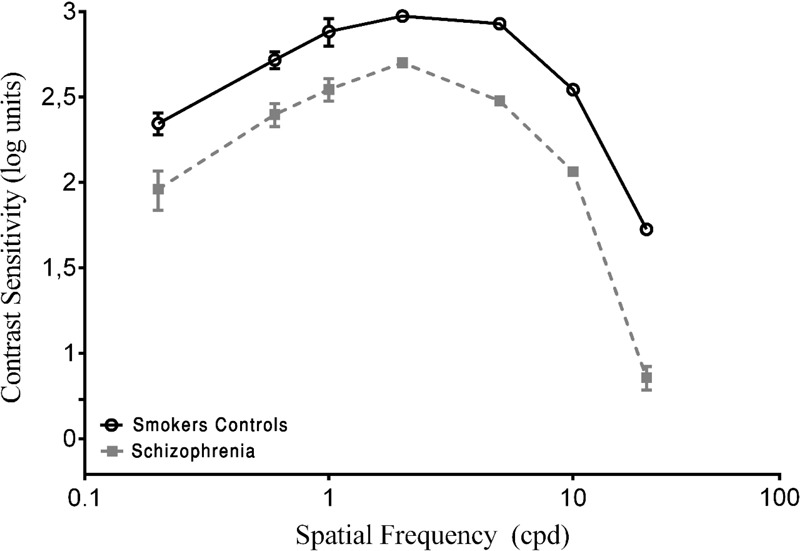
Contrast sensitivity curves as a function of spatial frequency cycles per degree (cpd) in smoker control group and schizophrenia groups. Each data point represents the mean sensitivity (reciprocal of contrast threshold). Error bars represent the standard deviation (SD) of the mean sensitivity. The contrast sensitivity function (CSF) is plotted in logarithmic units.

In the present study, the CSF is expressed in log units (reciprocal of contrast threshold). The results of the psychophysical measurements of contrast sensitivity are shown in **Figure 1**. The Kruskal–Wallis *H*-test indicated a significant difference in spatial frequencies between groups ($\chi_{2}^{2}$ = 59.53, *p* < 0.001, *w*^2^ = 0.76). The *post hoc* analysis with the Mann–Whitney *U*-test was performed with Bonferroni correction, resulting in a level of significance of *p* < 0.016. The median (semi-interquartile ranges – sIQR**)** for 0.2, 0.6, 1.0, 2.0, 5.0, 10.0, and 20.0 cpd were 84.18 (53–105), 143.16 (119–166), 180.18 (141–225), 221.72 (166–250), 183.82 (137–229), 110.98 (81–128), 36.55 (29–44), and 18.95 (12–31), respectively.

To obtain a baseline, both of the SCZ groups were compared with the SC group. A statistically significant difference in the CSF was found for all spatial frequencies (*p* < 0.001; **Figure 1**). The SCZ tobacco-nonuser group had lower sensitivity than the SC group (i.e., they needed more contrast to detect gratings) for all spatial frequencies (*p* < 0.001).

**FIGURE 2:**
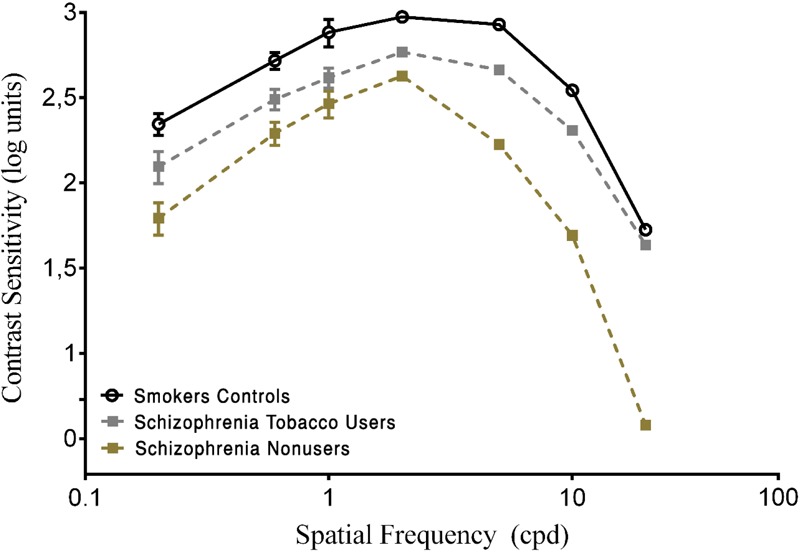
Contrast sensitivity curves as a function of spatial frequency (cpd) in the smoker control group and schizophrenia tobacco users group and schizophrenia non-users group. Each data point represents the mean sensitivity (reciprocal of contrast threshold). Error bars represent the standard deviation (SD) of the mean sensitivity. The CSF is plotted in logarithmic units.

Pairwise comparisons showed that the SCZ tobacco-user group was less sensitive than the SCZ tobacco-nonuser group for all spatial frequencies: 0.2 cpd (*U* = 54, *p* < 0.001, *r* = -0.62), 0.6 cpd (*U* = 66, *p* < 0.001, *r* = -0.57), 1.0 cpd (*U* = 58, *p* = 0.015, *r* = -0.60), 2.0 cpd (*U* = 69, *p* < 0.001, *r* = -0.56), 5.0 cpd (*U* = 44, *p* < 0.001, *r* = -0.67), 10.0 cpd (*U* = 64, *p* < 0.001, *r* = -0.58), and 20.0 cpd (*U* = 22, *p* < 0.001, *r* = -0.76). The SCZ tobacco-user group had larger CSF impairments than the SCZ tobacco-nonuser group (**Figure 2**).

### Color Vision

**FIGURE 3:**
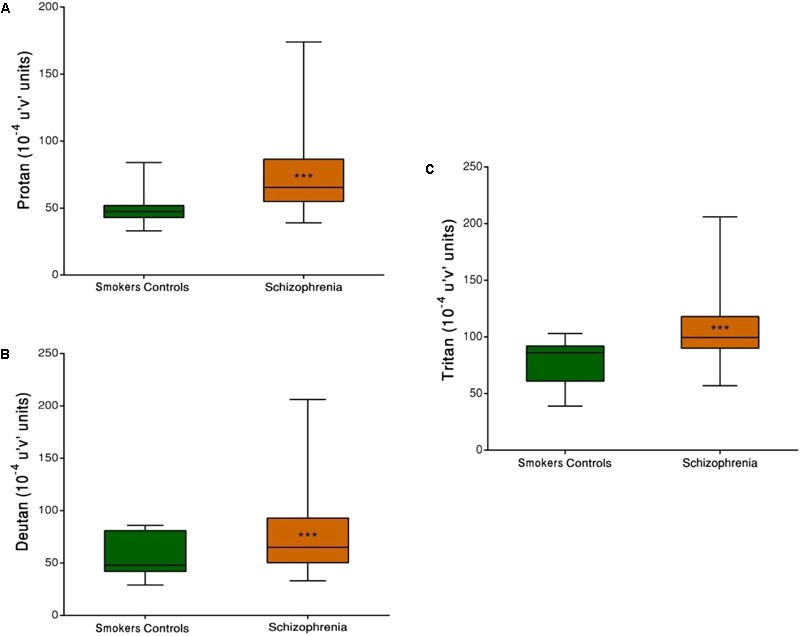
Trivector test, showing boxplots for protan **(A)**, deutan **(B)**, and tritan **(C)** confusion lines. The data are presented in 10^-4^ u’v’ units. Each boxplot is based on the results of 40 participants. ^∗∗∗^*p* < 0.001.

Both SCZ groups were compared with the SC group. Significant differences in discrimination thresholds along the three vector axes were found between groups (*p* < 0.001). The results of the Trivector measurements are shown in **Figure 3**.

**FIGURE 4:**
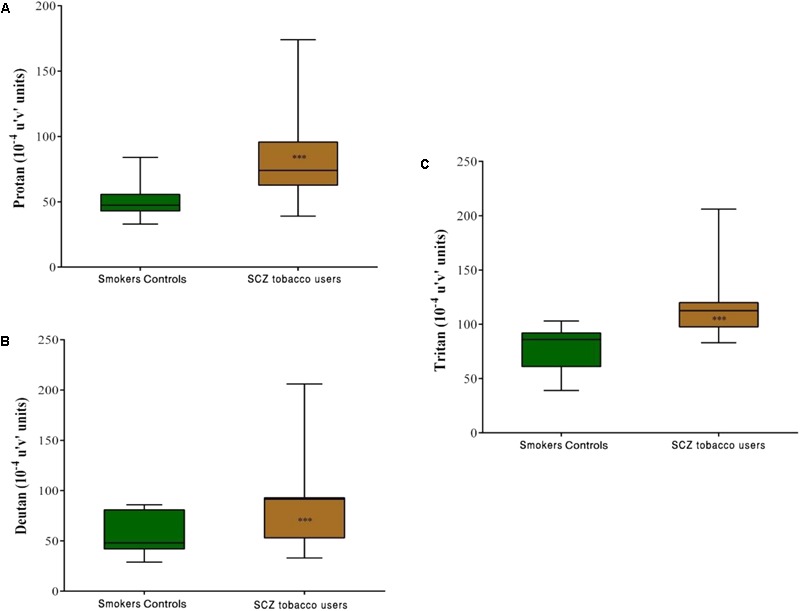
Trivector test, showing boxplots for protan **(A)**, deutan **(B)**, and tritan **(C)** confusion lines. The data are presented in 10^-4^ u’v’ units. Each boxplot is based on the results of 40 participants in the smoker controls (SC) group and 20 participants in the schizophrenia (SCZ) tobacco-user group. ^∗∗∗^*p* < 0.001.

Group comparisons revealed that the SCZ tobacco-user group had higher chromatic discrimination thresholds than the SC group along the protan axis (*U* = 61, *r* = -0.60, *p* < 0.001), deutan axis (*U* = 82, *r* = -0.52, *p* = 0.001), and tritan axis (*U* = 85, *r* = -0.51, *p* = 0.001; **Figure 4**).

**FIGURE 5:**
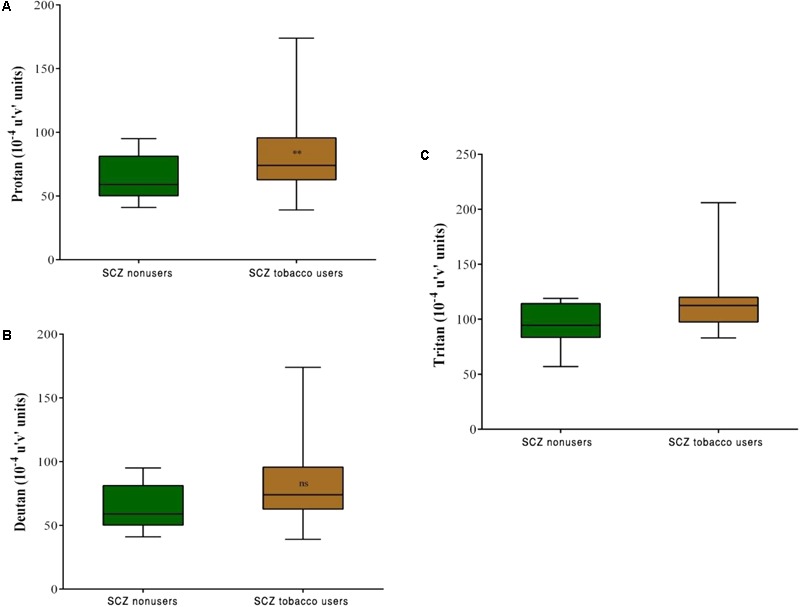
Trivector test, showing boxplots for protan **(A)**, deutan **(B)**, and tritan **(C)** confusion lines. The data are presented in 10^-4^ u’v’ units. Each boxplot is based on the results of 20 participants. ^∗∗^*p* < 0.01.

Comparisons between the SCZ tobacco-user and -nonuser groups revealed statistically significant differences in chromatic discrimination thresholds for the protan axis (*U* = 32, *r* = -0.45, *p* = 0.004), and tritan axis (*U* = 19, *r* = -0.54, *p* < 0.001), with no difference for the deutan axis (*U* = 75, *p* = 0.486; **Figure 5**).

### Clinical Measures and Visual Performance in Schizophrenia

The BPRS total score was correlated with all of the visual performance tests, except the tritan axis, among both SCZ groups, indicating that more severe symptoms at the time of testing was associated with worse visual processing performance (**Table 2**). No significant correlations were found between scales that assessed attention, memory, depression, or mania and the visual sensitivity tests (*p* > 0. 05).

**Table 2:** Correlation between visual measures and neuropsychological tests in the schizophrenia groups.

	LSF	MSF	HSF	Protan	Deutan	Tritan
BPRS	-0.75^a^	-0.74^a^	-0.58^a^	0.41^a^	0.40^a^	0.20
Stroop	0.13	0.19	0.08	-0.33	-0.09	-0.08
Flanker	0.11	0.14	0.19	-0.29	-0.19	-0.23
Hamilton	0.05	0.15	-0.20	-0.09	0.15	-0.11
YMRS	0.19	0.10	-0.05	0.23	0.43	0.41

*^*a*^p < 0.01. LSF, low spatial frequency; MSF, medium spatial frequency; HSF, high spatial frequency; BPRS, Brief Psychiatric Rating Scale; YMRS, Young Mania Rating Scale.*

Linear regression analysis was conducted to investigate possible effects of cognitive performance on visual sensitivity between groups. No significant predictors of visual processing were found in the Stroop Color-Word Interference, Flanker Task, Hamilton Scale for Depression and YMRS (*F*_3,76_ = 2.593, *p* = 0.060, adjusted *R*^2^ = 0.067, β = 0.030, *t* = 0.884, *p* = 0.380). Separate regression analyses indicated that BPRS scores were not predictive for contrast sensitivity in the SCZ tobacco-nonuser group (β = 0.330, *t* = 0.637, *p* = 0.527) for the protan (β = 0.026, *t* = 0.255, *p* = 0.799), deutan (β = 0.170, *t* = 1.870, *p* = 0.065), and tritan (β = 0.015, *t* = 0.181, *p* = 0.857) color confusion axes. A relationship was found between BPRS scores and contrast sensitivity in the SCZ tobacco-user group for the protan, deutan, and tritan color confusion axes (**Table 3**).

**Table 3:** Linear regression analysis of the relationship between Brief Psychiatric Rating Scale (BPRS) scores and visual task performance in the schizophrenia tobacco user group.

Dependent variable	Adjusted *R*^2^ of model	β	*t*-value	*p*-value
LSF	0.25	0.50	17.99	0.001^b^
MSF	0.10	0.20	11.11	0.001^b^
HSF	0.21	0.44	14.49	0.001^a^
Protan	0.38	0.62	14.60	0.001^b^
Deutan	0.05	0.26	5.45	0.05^a^
Tritan	0.09	0.27	5.84	0.05^a^

*^*a*^p < 0.001. ^*b*^p < 0.05. LSF, low spatial frequency; MSF, medium spatial frequency; HSF, high spatial frequency.*

## Discussion

The present study evaluated visual sensitivity in SCs compared with SCZ tobacco-user and -nonuser groups. The SCZ tobacco-user group had lower visual sensitivity than the SC group and SCZ tobacco-nonuser group for both chromatic and achromatic stimuli (**Figures 1, 3**). The CSF efficiently assessed possible effects of tobacco in SCZ patients, and these effects were modulated by low, medium, and high spatial frequency bands (**Figures 1, 2**). The CCT efficiently assessed discrimination thresholds for protan, deutan, and tritan axes (**Figures 3, 4**).

The CSF refers to the ability to detect variations in luminance. It is a relevant measure because of its low-level visual assessment, the impairment of which would suggest basic visual system disturbances in SCZ (Butler et al., 2008; Silverstein, 2016). Although it would be difficult to explain these disturbances in terms of cognition, a reduction of visual sensitivity could result from affective and cognitive deficits that are caused by the disease, such as illness severity, attention, working memory, planning, and psychomotor performance. We performed bootstrapping regression analysis to investigate this assumption. The results showed a relationship only between illness severity and visual performance, measured by the BPRS (**Table 3**). Investigating illness severity and illness duration may serve as a way to improve prognosis (Konarski et al., 2007). Non-response to treatment may lead to overall impairment, including a decrease in cerebral metabolism, which can cause atrophy in visual processing pathways (Wong-Riley, 2010).

With regard to color processing, trichromatic vision is based on the integration of short, medium, and long wavelengths by cone receptors. Another theory, the opponent process theory, implicates the existence of excitatory and inhibitory connections between cones (red–green and blue–yellow color vision (CV) systems; Goldstein, 2009). Both theories seek to explain optical and neural aspects of CV processing (DeValois and Webster, 2011). We observed a reduction of color discrimination thresholds in the SCZ group (**Figure 3**). More specifically, the SCZ tobacco-user group presented a significant reduction compared with the other two groups (**Figures 4, 5**), supporting the hypothesis that long-term smoking in schizophrenia may affect color vision.

Although the specificity of impairments that were observed in the magno- (M), parvo- (P), and koniocellular (K) pathways hinges on luminance conditions (Klistorner et al., 1997; Yoonessi and Yoonessi, 2011; Ahmadi et al., 2015), several authors concluded that spatiotemporal frequencies are associated with these pathways (Skottun and Skoyles, 2007; Cadenhead et al., 2013; Gracitelli et al., 2013; Skottun, 2015).

The dual channel model (involving the M- and P-pathway) is widely discussed in studies of visual psychophysics and schizophrenia. However, some studies reported an overall reduction of visual contrast sensitivity while isolating stimuli that would theoretically involve only one of these pathways (Skottun, 2000, 2015; Cadenhead et al., 2013). One may argue that this model is outdated and does not provide important descriptions of human visual function. Nonetheless, this model has provided insights into the function of chromatic and achromatic stimulus detection. For example, the M-pathway responds to achromatic stimuli with low spatial frequencies. The P-pathway responds to achromatic stimuli with high spatial frequencies and chromatic stimuli. Regardless of the luminance condition, both pathways can respond transiently, suggesting the importance of understanding these pathways separately to better understand overall function (Ahmadi et al., 2015; Skottun, 2015).

Under conditions that affect the central nervous system, such as SCZ and smoking, both the M- and P-pathways may exhibit dysfunction because of an imbalance of neurotransmitters (Kéri et al., 2002; Chen et al., 2003; Govind et al., 2009). For example, dopaminergic hypofunction that is caused by an imbalance of cortical upregulation may increase functional antagonism between the center and periphery of the receptive field of bipolar cells (Chaffiol et al., 2017; Nakao et al., 2018). The reverse may also be true, in which dopamine hyperfunction may decrease receptive fields of bipolar cells. Under light-adapted conditions, dopamine receptors are able to either decrease or increase the size of the receptive field (Zhang et al., 2011, 2012). Dopamine hyper- or hypofunction may involve changes that are related to spatial vision (Chen et al., 2003). Thus, abnormalities in these pathways may cause direct losses of the perception of stimuli at specific spatial or temporal frequencies. Nonetheless, there is a suggestion that an overall deficit of visual pathways may be more likely (Herzog and Brand, 2015).

Transient nicotine administration has been shown to improve cognition and visual backward masking (Levin et al., 1998; Heishman et al., 2010; Shaqiri et al., 2015), whereas long-term smoking may affect visual processing (Kunchulia et al., 2014; Fernandes et al., 2017a). The chronic use of cigarettes increases the expression of nAChRs in the nervous system (Besson et al., 2007), resulting in an imbalance of neurotransmission. Previous studies reported that chronic heavy smoking affected spatiotemporal vision in smokers (Kunchulia et al., 2014; Fernandes et al., 2017a). Therefore, in the present study, we opted to not include a group of healthy controls because this would not provide novel insights into this assumption.

As widely reported in the literature, there is a relationship between genetic variations of the nAChR α7 subunit (CHRNA7) and SCZ (Bakanidze et al., 2013). The deletion of 15q13.3 (i.e., the location of CHRN7 on the chromosome) may directly impact post-synaptic nAChRs (Deutsch et al., 2016). nAChRs are composed of α2–α10 and β2–β4 subunits, and one genetic variation of CHRNA7 may affect the response to chronic smoking (Dani, 2015). Furthermore, nAChRs are involved in modulating neurotransmission. Thus, the nicotinic system plays an important role in chronic smoking that is observed in SCZ patients. Genome-wide association studies of SCZ reported higher rates of smoking but failed to explain how this occurs (Vink et al., 2009; Gage et al., 2017). The function of the CHRNA5–A3–B4 cluster suggests a relationship between higher rates of smoking in SCZ patients (Lassi et al., 2016). A recent study reported evidence of a non-causal effect of tobacco use in SCZ (Lassi et al., 2016). The lack of a causal relationship between higher rates of smoking and schizophrenia may be related to the minor allele of the rs1051730 single-nucleotide polymorphism of the CHRNA5–CHRNA3–CHRNB4 region (Wium-Andersen et al., 2015). The aforementioned studies provided the impetus for conducting the present study.

The use of medications in SCZ seeks to improve disease symptoms. Pharmacological treatments usually involve antipsychotic medications, the function of which is to reduce psychotic states, decrease hallucinations, and improve symptomatology through the blockade of dopaminergic neurotransmission in the mesocortical and mesolimbic pathways (Lally and MacCabe, 2015). Typical antipsychotic drug treatment can impair visual processing. Several studies reported that atypical antipsychotics improved visual processing compared with typical antipsychotics (Chen et al., 2003; Cadenhead et al., 2013; Shoshina and Shelepin, 2015).

To homogenize the groups in the present study, only patients who took atypical antipsychotics were considered (**Table 1**). The CSF has been shown to decrease as the affinity of a drug class for dopaminergic neurotransmission increases (Chen et al., 2003; Cadenhead et al., 2013). In the case of atypical antipsychotics, dopaminergic affinity is lower. We employed visual psychophysics to determine whether the effects of smoking would be more evident than the effects of antipsychotic drugs. We found that smoking further affected contrast sensitivity beyond the effects of antipsychotics.

The SCZ tobacco-nonuser group was less severely impaired than the SCZ tobacco-user group, which may explain why these SCZ patients did not smoke. The SCZ tobacco-nonuser group also had better visual sensitivity. One limitation of the present study, however, was that the SCZ patients were not grouped according to illness duration. Chronic tobacco use in SCZ increases the activity of enzymes (e.g., CYP 1A2) that decrease the concentrations of antipsychotics (Sagud et al., 2009). According to Chen et al. (2003), the lack of use of medication would decrease contrast detection thresholds, thus increasing contrast sensitivity. The chronic use of cigarettes may decrease the concentrations of medications in the body. Therefore, the deleterious effects of antipsychotic on CSF may be significantly influenced by cigarette use.

One may argue that smoking affects vision, causing pupillary constriction (Lie and Domino, 1999), and our findings may be explained in view of this. However, the tests we conducted in the present study were performed at a relatively close distance. That is, for stimuli that were near the observer, pupillary constriction would still allow sufficient light energy distribution to detect the stimulus. Under photopic conditions, such as in the present study, and using tasks in which the observer is close to the computer monitor, pupillary constriction occurs automatically. Thus, even if cigarette smoking causes pupillary constriction, the relative energy that is necessary for detecting the stimulus is sufficient under photopic conditions (Alfonso et al., 2007). Schizophrenia may also be correlated with a decrease in pupillary diameter, which can affect the detection of complex stimuli (Minassian et al., 2004). However, the stimuli that were used in the present study were elementary in terms of visual processing and required minimal cognitive activity. Moreover, our regression analysis did not reveal a significant effect of cognition on visual performance. Nonetheless, we did not evaluate pupillary diameters pre- vs. post-smoking, which may be another limitation of the present study.

Our study replicates previous findings about impairments in visual processing in SCZ (Skottun and Skoyles, 2007; Cadenhead et al., 2013; Plomp et al., 2013; Silverstein and Rosen, 2015) and provides new directions for research on SCZ, tobacco use, and spatial vision. One promising approach could be to administer nicotine directly to SCZ tobacco-nonusers (e.g., using nicotine patches) to assess the CSF and other visual functions. Our study also highlights the need to evaluate nicotine-addictive behavior in patients with SCZ. Overall, our findings demonstrate the consequences of nicotine on the visual system in patients who are diagnosed with SCZ.

Despite the novelty of the present study, it also has several limitations. First, the findings would have been bolstered by specific measures of nicotine intake (e.g., serum cotinine and breath carbon monoxide). However, we attempted to control some variables in this regard, so we believe that this limitation did not affect our overall results. Second, the sample size may not have been sufficient to obtain reliable results in the regression analysis. Although a standard in psychophysical and perceptual studies, our sample size may be a limitation. Third, we cannot ascribe the differences that we observed to any specific visual pathway. Fourth, we did not randomize or evaluate the SCZ patients based on illness duration. Fifth, we used Snellen’s eye chart solely as a screening tool and not as an additional way of investigating visual acuity, which may also be a limitation. Our future studies will use logMAR rather than the eye cart as a screening tool. Sixth, the use of the BPRS instead of the Positive and Negative Symptom Scale (PANSS) may also be a limitation. However, we sought to verify whether the severity of SCZ symptoms, based on the BPRS, is a predictor of visual sensitivity. We will employ the PANSS in future studies.

The present findings need to be tested in clinical trials or larger controlled studies. The present study did not reveal a definitive origin of the losses in visual sensitivity. We employed a systematic methodology to evaluate visual sensitivity thresholds through the application of standardized tests with adequate levels of sensitivity and specificity. Such tests have been employed in previous studies of spatial vision.

In summary, the present results indicated significant differences in visual sensitivity in SCZ patients who were chronic tobacco users. Our findings justify future research on the pathophysiological mechanisms that are involved in these sensorial alterations.

## Author Contributions

TF: conceived and designed the experiments, participated in its coordination, performed the statistical analysis, and helped draft the manuscript. MA: helped revise the manuscript and interpret the data. JS: helped interpret the data and perform the statistical analysis. RN: conceived the experiments, helped interpret the data, and provided the statistical tools. NS: conceived and designed the experiments, helped draft the manuscript, and helped interpret the data. All of the authors read and approved the final manuscript.

## Conflict of Interest Statement

The authors declare that the research was conducted in the absence of any commercial or financial relationships that could be construed as a potential conflict of interest.
